# TOWARDS AN UNDERSTANDING OF DISTURBED SLEEP PHENOTYPES AFTER TRAUMATIC SPINAL CORD INJURY

**DOI:** 10.2340/jrm.v58.44651

**Published:** 2026-03-17

**Authors:** Letitia Y. GRAVES-DIXON, Anna MAY, Susan REDLINE, Zixiang XU, Jiayang SUN, Adam R. FERGUSON, Kath M. BOGIE

**Affiliations:** 1 School of Nursing, University of Texas Medical Branch, Galveston, TX; 2 Louis Stokes Cleveland VA Medical Center, Cleveland, OH; 3 Harvard University, Cambridge, MA; 4 George Mason University, Fairfax County, VA; 5 University of California San Francisco, San Francisco, CA; 6 San Francisco Veterans Affairs Medical Center, San Francisco, CA; 7 Case Western Reserve University, Cleveland, OH, USA

**Keywords:** heart disease, metabolic disease, sleep hygiene, spinal cord injuries, Veterans’ health

## Abstract

**Objective:**

Examine the Spinal Cord Injury-Pressure Injury Resource (SCI-PIR) database to assess the prevalence and identify relationships among sleep disorders and cardiometabolic risk after spinal cord injury.

**Design:**

Retrospective observational cohort study using the Department of Veterans Affair SCI-PIR database.

**Subjects/Patients:**

18,894 Veterans living with spinal cord injury.

**Methods:**

The SCI-PIR database was queried for ICD9 codes related to cardiovascular, metabolic, psychological, and sleep conditions to identify subgroups of spinal cord injury individuals with sleep disorders and associated clustering of cardiometabolic risk factors and sleep diagnoses. Multiple correspondence analysis probed the underlying associations. Cramer V statistics confirmed and quantified the associations.

**Results:**

Sleep apnoea (6.7%) and insomnia (4.3%) were the most common sleep diagnoses. Multiple correspondence analysis demonstrated 2 phenotypic clusters: Cluster A showed robust links between sleep apnoea, hypersomnia, heart failure, and arrhythmias, and secondary associations with coronary artery disease, chronic kidney disease, obesity, diabetes, and hyperlipidaemia. Cluster B showed strong relationships between insomnia, anxiety, and post-traumatic stress disorder.

**Conclusion:**

2 distinct sleep clusters were identified for persons with spinal cord injury. This analysis supports previous findings that sleep disorders associate with overall health in individuals with spinal cord injury, and particularly cardiovascular health. ICD9 coding may under-report sleep diagnoses. Data-driven statistical analysis can uncover insights into the complex interplay between spinal cord injury and secondary health conditions.

## LAY ABSTRACT

This study looked at how sleep problems relate to heart and metabolic health in Veterans with spinal cord injuries. Using a large database from the Department of Veterans Affairs, researchers analysed health records of nearly 19,000 Veterans with spinal cord injuries. We found that sleep apnoea and insomnia were the most common sleep issues. Two distinct groups emerged: 1 linked sleep apnoea and excessive sleepiness with heart failure and irregular heartbeats, along with other conditions like diabetes and obesity. The other group showed a strong connection between insomnia, anxiety, and post-traumatic stress disorder. These findings suggest that sleep problems in people with spinal cord injuries are closely tied to overall health, especially heart health. The study also highlights that sleep issues may be underdiagnosed, and that using data-driven methods can help uncover important health patterns.

While precision health continues to demonstrate value in providing the right diagnoses and interventions to the right patient at the right time, precision rehabilitation lags (1). Spinal cord injury (SCI) is the leading cause of paralysis in the USA after stroke and is characterized by chronic systemic inflammation, multisystem perturbations, and widespread disability (2, 3). For more than 90% of people living with SCI, multimorbidity, defined as 2 or more chronic co-occurring secondary health conditions (SHCs), is all too common. Individuals with complex interacting SHCs require additional ongoing management and increased healthcare utilization, often with worse health outcomes (4). Disturbed sleep is one of the most significant SHCs and is a known risk factor causing or exacerbating comorbidities, particularly those impacting cardiometabolic health, as indicated below. Evidence suggests that individuals living with SCI have a two- to four-fold higher prevalence of sleep disorders compared with the non-injured population, yet they remain under-diagnosed and under-treated (5). Individuals with SCI suffer from worse self-rated sleep quality, together with cardiovascular autonomic dysfunction and neuroendocrine changes that impact control of breathing and sleep–wake regulation (5, 6). Sleep disturbances encompass disorders of breathing such as apnoea, insomnia, hypersomnia, parasomnia, movement, and circadian rhythm (6). Treatment of these sleep disorders can provide a key target for improving cardiometabolic health and overall wellness, especially in this vulnerable population.

Sankari and colleagues (2017) found that “sleep disturbance and co-morbidities have a bidirectional relationship that requires a comprehensive approach to address sleep and chronic conditions simultaneously” (5). A multiplicity of risk factors may influence sleep disease detection, assessment of disease severity, phenotypic classification, and treatment response (7). Phenotyping is a critical methodological approach for characterizing heterogeneity within clinical populations, particularly in complex conditions such as SCI. SCI presents with diverse physiological, neurological, and behavioural manifestations, complicating both research and clinical management. In this context, phenotyping enables the identification of distinct subgroups based on shared clinical features, thereby improving diagnostic precision, informing treatment strategies, and enhancing the interpretability of research findings.

This study covered the associations between various sleep disorders and cardiovascular diseases, as well as relationships among sleep disorders and among different levels of cardiovascular risk factors and conditions. Therefore, they are useful to identify individuals with SCI who are at elevated risk of sleep disorders and associated cardiometabolic morbidity and mortality. The creation of this grouping/clustering has the potential to support both clinical decision-making and research by enabling more targeted (sub-)categorization or assessment of comorbidity, diagnosis, and intervention.

Identifying sleep phenotypes in SCI is not only methodologically innovative but also clinically significant. In this study, we leveraged data from the Spinal Cord Injury Pressure Injury Resource (SCI-PIR) database to derive data-driven representations of sleep disorder patterns in individuals with SCI (hereafter referred to as *computational phenotyping*) to enhance cohort stratification and support downstream precision health initiatives. The electronic health record (EHR) provides a valuable platform for this work, offering access to a wide array of phenotypic features such as sleep metrics, cardiometabolic indicators, and clinical comorbidities (8). By applying multiple algorithmic approaches, we sought to determine the most effective methods for computational phenotyping and subgroup grouping/clustering.

This study represents a foundational step towards integrating computational phenotyping in SCI research. By characterizing the prevalence and associations of sleep disorders using EHR-derived data, we seek to lay the groundwork for a future framework that supports future epidemiological and translational investigations. Ultimately, this work will contribute to a deeper understanding of the mechanisms linking sleep and cardiometabolic health in SCI and support the development of targeted, data-driven interventions.

## METHODS

**Fig. 1:**
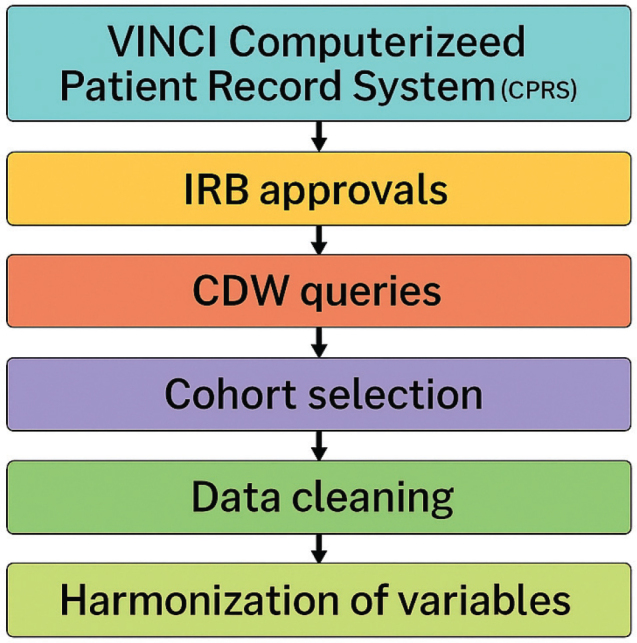
Data extraction workflow.

This retrospective observational cohort study used population-based data from the Department of Veterans Affair SCI-PIR database (9) containing comprehensive clinical data collected from more than 120,000 encounters between September 2010 and September 2015 for 29,000 individual Veterans with SCI. SCI-PIR data include clinical characteristics of Veterans with SCI, demographics, and comorbidities identified as being relevant to sleep and cardiometabolic risk. We queried the database for ICD9 diagnosis codes related to cardiovascular, metabolic, psychological, and sleep conditions to identify sleep disorder subgroups defined by clustering of cardiometabolic risk factors and sleep diagnoses for individuals with SCI diagnosed with sleep disorders. The workflow for data extraction is shown in Fig. 1.

### VINCI computer data warehouse (CDW)

To interrogate national VA data, we used the VA Informatics and Computing Infrastructure (VINCI) – a secure workspace facilitating data access and providing analysis tools. Data were de-identified and fully anonymized. Institutional Review Board (IRB) approval was obtained through the VA Northeast Ohio System (VANEOHS) Research Office for creation of the original database. The requirement for informed consent was waived.

Through VINCI, we accessed CDW databases to extract patient data. The CDW includes data related to approximately 9 million Veterans across all 50 states. Available data include, but are not limited to, International Classification of Disease-Clinical Modification (ICD-CM) codes (9th and 10th revision), and International Classification of Disease-Procedural Classification System (ICD-PCS) codes (9th and 10th revision), as well as demographic data and data from the inpatient/outpatient EHRs and inpatient/outpatient pharmacies. The Workspace is provisioned so that each study has its own project site where multiple people can collaborate using a common set of software tools and files.

ICD-9 codes from CDW were used as phenotypic features to identify relationships among sleep disorders and cardiometabolic health and provide the foundation for their use in targeted treatment and management. The reason for selecting ICD-9 codes instead of the ICD-10 codes, was twofold: (*i*) the parent study used ICD-9, which at the time pre-dated ICD10 development, (*ii*) the increased number of diagnosis codes from ICD-10 for each condition was not to our benefit for SCI diagnoses.

### Study participants

Criteria for inclusion were Veterans who sustained a traumatic SCI and received SCI care in the VA healthcare system. This study used the SCI-PIR database, an existing intramural dataset (9, 10) developed from the VINCI Corporate Data Warehouse (CDW). Extracted datasets include all SCI diagnostics, demographics, comorbidities, and medications collected. Commonly reported symptoms were quantified and characterized by demographic and injury characteristics, and frequency of co-occurrence using the SCI-PIR. Data of interest to the current study are summarized in Table I.

**Table I:** Data of interest to the current study

Abbreviation	Diagnosis/condition	ICD-9 code
Spinal cord injury diagnosis		
Tetra	Tetraplegia	344.0
Para	Paraplegia	344.1
SCINos	SCI not otherwise specified	952.9
Cardiac conditions		
AF	Atrial fibrillation	427.31
HF	Heart failure unspecified	428.9
HTN	Hypertension	401
Arr	Arrhythmia	427.9
CAD	Coronary Artery disease	36.11–36.14
Sleep conditions		
INSM	Insomnia	780.52
HSN	Hypersomnia	327.1
OSA	Obstructive sleep apnoea	327.23
Sapn	Sleep apnoea (mixed type)	327.2
Psychological conditions		
Smok	Tobacco	305.1 989.84
Dep	Depression	V79.0
DU	Drugs use	305.2–305.7
Psycs	Psychosis	298.0–298.8
PTSD	Post-traumatic stress disorder	309.81
ETOH	Alcohol use	305.0
Anx	Anxiety	300.0
Metabolic conditions		
HLD	Hyperlipidaemia	272.0–272.4
DM	Diabetes mellitus	250
HTh	Hypothyroidism	244.9
LDZ	Liver disease	571
OBE	Obesity	V77.8
CKD	Chronic kidney disease	585.3–585.6

### Statistics

**Fig. 2:**
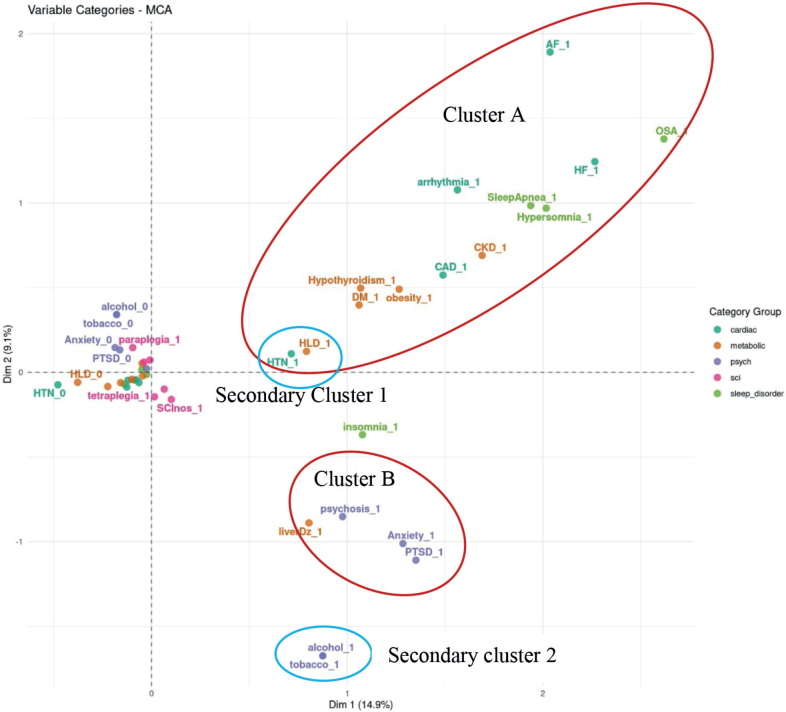
Multiple correspondence analysis of variable categories clusters.

**Fig. 3:**
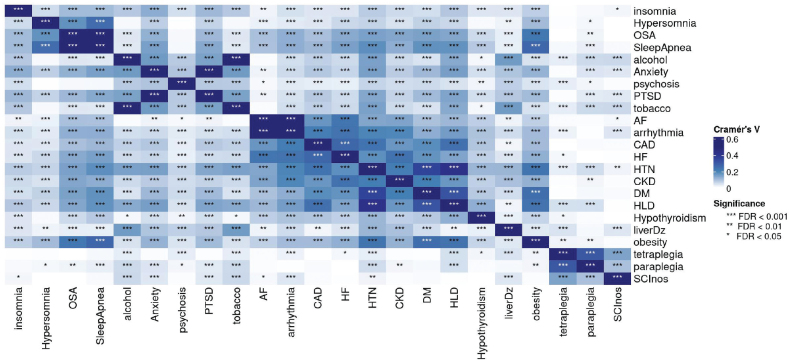
Level of significance based on the Cramer’s V values.

**Fig. 4:**
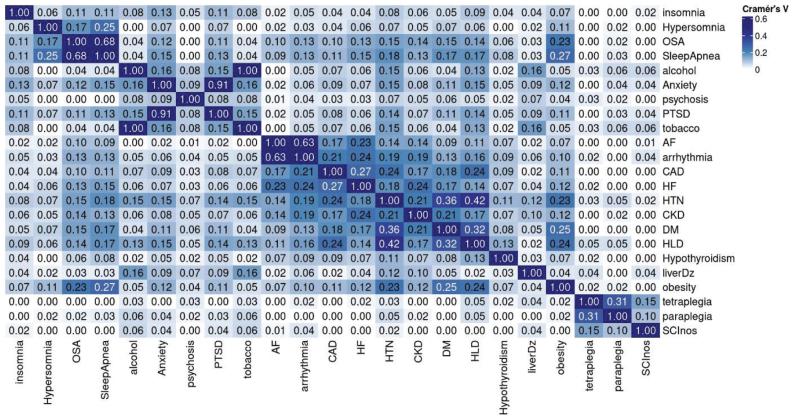
Cramer’s V values.

The data on these ICD9 codes allowed computation of the disease diagnosis occurrence and co-occurrence, as well as counts for demographics, to form a multiway contingency table of categorical variables of interest. Multiple correspondence analysis (MCA) of the multiway contingency table provides a powerful method for analysing the underlying structures of such categorical data (11). MCA transforms each level of categorical variable, such as the categorial variable name “Sleep Conditions” in Table I, into a point in a geometric space as shown in Fig. 2, facilitating clustering analysis of these categories within and between variables. The interpretation of an MCA plot follows 2 key principles. First, the closer the categories (or the points) are in the MCA plot, the stronger their associations. Second, groups of points with similar closeness but further from the origin in the MCA plot represent stronger relationships than the points with similar closeness that are crowded at the origin. Phenotypic clusters or groupings of points away from the origin in an MCA plot help visualize whole relationships within and between categories and identify patterns in the data, without defining outcome variables. We therefore used MCA to help identify associations and clusters among the comorbidities listed in Table I. We also further quantified these association in the effect size and significance, using the Cramer V statistic in Figs 3 and 4.

## RESULTS

As shown in Tables II and III, the cleaned cohort totalled 24,118 Veterans and was predominantly male (96%) and white (69%). 5,224 individuals were excluded from the current analysis because they did not have a clear diagnosis of any sleep disorder. In the sub-set of 18,894 Veterans who had explicit diagnosis information (Yes or No in at least 1 category of each variable), sleep disturbances occurred in only 16.14%, with sleep apnoea (6.7%) and insomnia (4.3%) being the most common sleep diagnoses.

**Table II:** Study population demographics

Factor	*n*	% of total population
Male	23,042	96
Female	1,070	4
Tetraplegia	7,500	3
Paraplegia	9,072	38
Sci, unspecified	6,141	25
Poorly categorized	~1,400	6
Married	11,867	49
Divorced	6,397	27
Never married	3,581	24
American Indian or Alaska native	136	0.7
Asian	100	0.6
Black or African American	4,063	21.5
Multiracial	363	1.9
Native Hawaiian or other Pacific islander	184	1.0
White	14,046	74.3
White not of Hispanic origin	2	0.0

**Table III:** Prevalence of sleep disorder

Disorder	Counts (out of 18,874 total)	% of total population (count/18874)	% of sleep disorder patients (count/3050)
Insomnia	818	4.33	26.82
Hypersomnia	233	1.23	7.64
Obstructive sleep apnoea	608	3.22	19.93
Sleep apnoea mixed	1,260	6.67	41.31
Unspecified sleep disorder	131	0.69	4.30
Any sleep disorder	3,050	16.14	100.00

*The group “unspecified sleep disorder” refers to participants who reported sleep disorder that does not fall into the 4 disorder cases (insomnia, hypersomnia, obstructive sleep apnoea, sleep apnoea mixed).

Note that the summation of “No” and “Yes” responses in each row is 18,894. Some individuals had multiple types of sleep disorders. “Any Sleep Disorder” includes patients with at least 1 of the 4 identified disorders, and those with an unspecified sleep disorder.

MCA-based computational phenotyping of sleep, cardiac disorders, and other comorbidities of 18,894 Veterans with complete information is given in Fig. 2 and demonstrates 2 phenotypic clusters. The first cluster (Cluster A) showed a robust primary link between sleep apnoea, hypersomnia, heart failure, and arrhythmias, and secondary associations (closer to the origin than the primary link) with coronary artery disease, chronic kidney disease, obesity, diabetes, and hyperlipidaemia, while hypertension and hyperlipidaemia are also close to each other, showing a natural chain effect. The second cluster (Cluster B) showed a strong relationship between insomnia, anxiety, and post-traumatic stress disorder, while hypertension is closer to Cluster A than to Cluster B. Circadian dysregulation, central sleep apnoea, restless leg syndrome, and hypersomnia had insufficient data from the EHR and were thus dropped from further analysis. Figs 3 and 4 show the statistical significance and the effect size (or strength) of associations between pairs of conditions, measured using Cramer’s V, ranging from 0 to 1. Values in the ranges (0, 0.1), (0.1, 0.2), (0.2, 0.4), (0.4, 0.6), (0.6,1) indicate negligible, weak, moderate, relatively strong, and strong associations, respectively. Significance is measured by the false discovery rate (FDR). The smaller the FDR, the more significant the Cramer V is from zero.

## DISCUSSION

The current study supports previous findings that sleep disorders following SCI are strongly associated with overall health, particularly cardiovascular health. Using structured EHR data, we identified 2 distinct sleep clusters (see Fig. 2). In persons with SCI, Cluster A demonstrates a strong link between sleep apnoea and cardiometabolic conditions. Cluster B indicates a strong relationship between insomnia and mood/psychological conditions. Sleep in the non-SCI population has been linked to several comorbidities that impact duration and quality of life, e.g., sleep apnoea to cardiovascular mortality – and could serve as an important marker of health status (12–16). It is also recognized that interactions between distinct co-occurring diseases further complicate diagnosis and treatment, and impact clinical decision-making. SCI contributes to a significant number of risk factors that predispose individuals to respiratory compromise, altered peripheral circulation, impaired immune regulation, and mood disturbances, all key factors in the development of sleep-disordered breathing, circadian sleep–wake disorders, and insomnia disorder (6). The ability to implement prognostic and diagnostic tools in the management of SCI could be an important first step in decreasing multimorbidity and increasing quality of life for persons with SCI.

The SCI literature is clear that there are high rates of sleep-disordered breathing and other apnoea-related conditions (6, 17, 18). Furthermore, our findings confirm that while disturbed sleep remains an underdiagnosed SHC after SCI, specifically in Veterans, most clinical research has focused on sleep apnoea and thus it is more readily recognized and screened for. A systematic review and meta-analysis by Graco et al. (2021) demonstrated the prevalence of sleep-disordered breathing in people with a tetraplegia level of injury at 83% (17). However, 2024 conference proceedings by Furlan showed that in SCI sleep disorder research only 4 studies focused on insomnia (18). Our study also illustrates that sleep diagnoses may be under-reported in this population.

Insomnia profiles, and associated exacerbating or secondary conditions, are not well characterized. Insomnia itself is a heterogeneous disorder; however, a symptom cluster approach can offer more targeted, patient-centred management of relevant symptoms (19). A psychoneurological cluster is characterized by emotional and/or behavioural symptoms that may be related to psychological and/or neurological dysfunction (20). This often manifests clinically as depressive symptoms, cognitive disturbance, fatigue, sleep disturbance, pain, and emotional distress (21). Several studies have linked insomnia to depression and anxiety. Specifically, in Veterans with SCI, insomnia severity was linked to depression, anxiety symptom severity, and risk of PTSD (21, 22).

To our knowledge, this is the first study to demonstrate SCI-specific phenotypic clusters with a distinct psychoneurological cluster related to symptoms of insomnia. However, further research is needed to refine these cluster models for clinical translation and application. In particular, there is a gap in sleep research focused on insomnia in persons with SCI.

Symptom cluster research has been influential in advancing our understanding of disease and treatment-related symptoms, particularly in oncology patients. A symptom cluster is defined as a group of stable, concurrent, and distinct symptoms that may share a common aetiology, mechanism, outcome, or variance (23 24, 25). Crawford et al. used a symptom cluster approach in their study of insomnia and the effort to develop more person-centred precision approaches to treatment (19). In their study, they utilized latent profile analysis, which looks at patterns of interrelatedness with the aim of targeting within-class homogeneity and between-class heterogeneity. It is important to note that while the authors demonstrate positive findings with their analysis, this is 1 of only 3 sleep-focused symptom cluster studies.

Across all symptom cluster literature, sleep or otherwise, there is no consensus on the best analytic methods for analysis; however, principal component analysis, hierarchical cluster analysis, and other variations of clustering approaches have been often used for numerical data clustering (26–28). A review by Khan et al. (28) demonstrated that clinical prognostic models, or statistical rules relating a desired outcome to 1 or more predictor variables using machine learning, are critical for machine learning to become a more mainstream tool in SCI.

### Limitations

One of the major limitations encountered was the large number of diagnostic codes for SCI and diagnosis codes that were no longer applicable but had not been deactivated in the EHR. For example, if a patient was initially classified as having tetraplegia based on the International Standards for Neurological Classification of Spinal Injury (ISNCSCI) at the time of injury but later was determined to have incomplete paraplegia, the former diagnosis code was not deactivated, showing the patient as having both tetraplegia and paraplegia. This ultimately led to the exclusion of approximately 6,900 individuals from the study cohort. It was helpful to have clinicians on the study team to assist with these clinical nuances; however, with large datasets such as this, the feasibility of hand-sorting cases is limited. Good quality source data is critical for quality results and is essential for effective automation. As we move toward precision medicine, more precise variables and electronic reminders to deactivate or remove or transform outdated ICD codes will improve the use of this structured data in the research domain.

Although health records are a treasure trove of clinically relevant data, EHRs are primarily designed to be used for billing and coding, making data challenging to work with for research applications. EHR data require proper cleaning, defining cohorts, and potentially redefining variables for clinical relevance (29) to avoid the “garbage in, garbage out” phenomenon. There are 2 main types of data: structured data, such as ICD codes and lab values, and unstructured data, including text data, such as physician notes (30). Historically, structured data have been the focus of computable clinical phenotyping efforts. However, keywords from unstructured text notes also hold key clinical information that can improve how patient cohorts are identified and clinical phenotypes are defined, and guide treatment plans and development (31). The combined use of structured and unstructured data will improve operationalization and generalizability.

### Conclusion

Sleep disturbances are common but underdiagnosed in Veterans with SCI. Our findings support the link between medical complications and the consequences of disturbed sleep and cardiometabolic risk. Our findings also suggest the benefits of data-driven statistical models in better understanding the interplay between complex conditions such as SCI and secondary health conditions. This lays a foundation for SCI-specific phenotyping, which can support the development of diagnostic and risk biomarkers, assessing the likelihood of developing sleep disorders, leading to the development of targeted interventions and treatments to advance precision health for Veterans and civilians with SCI.
